# Remission of aggressive autoimmune disease (dermatomyositis) with removal of infective jaw pathology and ozone therapy: review and case report

**DOI:** 10.1007/s13317-018-0107-z

**Published:** 2018-06-30

**Authors:** Robert Jay Rowen

**Affiliations:** Private Medical Practice, 2200 County Center Dr. Ste C, Santa Rosa, CA 95403 USA

**Keywords:** Ozone therapy, Endodontically treated teeth, Cavitation, Autoimmune disease, Inflammation, Dermatomyositis, Infection, Immune modulation, Antimicrobial, Antibacterial

## Abstract

**Introduction:**

This case uniquely reports a connection between endodontically infected teeth and systemic disease, and additionally presents ozone therapy as a unique therapy and immune system modulator. It is the world’s first such reported case and the treatment holds invaluable lessons in assessing the “unknown” causes of autoimmunity and inflammation. Additionally, it presents ozone therapy as a most needed unique, non-toxic and powerful anti-infective agent, anti-inflammatory and immune modulator.

**Case presentation:**

The patient was a Mexican male field laborer, age 48 years, in inflammatory crisis with a confirmed case of dermatomyositis. He had received massive prednisone, and powerful immune suppressing drugs just to function, while disease still raged. I encountered him in the field in June 2012 with severe muscle pain, weakness, and diffuse generalized skin rash, essentially unable to do his work. Creatine kinase peaked at 9293 U/L. History and physical examination findings caused suspicion of subclinical infections in endodontically treated teeth. This impression was confirmed in subsequent dental evaluation. He fully recovered after dental infections were confirmed and surgically removed, while receiving ozone therapy until all symptoms and laboratory abnormalities normalized.

**Conclusion:**

Dental focus of occult infection may be a prime cause/trigger of autoimmune disorders and inflammatory disorders, requiring surgical intervention to remove. Ozone therapy, little known in conventional medicine, has been shown in the literature and in this case to be a powerful and safe immune modulator and anti-infective agent. This case has significant relevance across the entire spectrum of both medical and dental practice. It also emphasizes the need for individualized assessment and treatment rather than symptomatic pharmacological approaches treating a “disease” rather than the patient. Subclinical dental infection and ozone therapy are reviewed.

## Introduction

Dermatomyositis is a disease belonging to the rheumatologic class, long considered an “autoimmune disease” of idiopathic cause. New research is now linking “autoimmune” and other diseases to heretofore unknown occult infection arising from or translocating from the mouth [1] and alimentary canal [2]. This disease is characterized especially by rash, weak muscles (especially proximal), and muscular atrophy. Creatine kinase enzyme may be extremely high, indicating muscle damage. Standard conventional treatments may be harsh. Drug therapy includes corticosteroids, immunosuppressants, antimalarials, and cytotoxic drugs. It has a 45% survival rate after 9 years [3]. There are reports of “spontaneous remission” of the disease in mild cases, but none of aggressive cases. This case is unique in that it is the first to present a remission of an aggressive case addressing endodontically infected teeth and the use of a little known therapy—ozone. It has relevance to the entire spectrum of immunology, rheumatology, and infectious diseases.

Ozone is an allotrope of oxygen, O_3_, the strongest naturally occurring oxidant, generated by lightning and ultraviolet irradiation. Medical ozone is made similarly. Oxygen passes through a high voltage corona arc discharge. Medical “ozone” is actually a mixture of oxygen (95–99%) and ozone (1–5%).

Ozone has been in continuous medical use for over 100 years, used by German soldiers in the trenches to disinfect wounds in WWI. Recent research both in vitro and in vivo demonstrate ozone to have powerful immune modulating and healing effects. In addition, it virtually instantly destroys bacteria [4, 5] and viruses some 150× faster than bleach, another oxidant.

Modern methods of ozone delivery include a direct intravenous (DIV) gas protocol, rectal insufflation of gas, and major autohemotherapy method (MAH) developed by Germans. In MAH, an aliquot of blood is removed from the body, heparinized, treated with O_2_/O_3_ (“ozone”) gas mixture, and returned.

Direct intravenous oxygen gas has been long used in medicine. Regelsberger reported it provides powerful immunological effects, and improves blood rheology and oxygenation [6]. It also increases the important prostacyclin/thromboxane ratio [7]. Robins has successfully used DIV oxygen/ozone gas mixture (up to 55 mcg O_3_/cc) for nearly 30 years and developed a protocol for administration [8].

In the form of MAH used herein, an aliquot of venous blood is removed from the patient (~ 200 cc at a time) in a vacuum glass bottle. Oxygen/ozone gas mixture, 200 cc at 70 mcg/cc ozone, is pumped into the bottle and swirled, mixing the gas with blood. Pressure in the bottle is maintained at 1.9–2.0 ATA (atmospheres absolute) by pumping additional O_2_ and the blood then returned to the patient under this pressure. A single pass of 200 cc blood plus gas delivers 14,000 mcg O_3_. A “double pass” would deliver 28,000 mcg O_3_ into 400 cc blood. This form of MAH is called “hyperbaric ozone” (HBO_3_) therapy.

## Case presentation

The patient, a muscular Mexican field hand, husband and father, was 48 years old when I saw him attempting to work on 12/6/2012 looking extraordinarily ill. He had exceptionally severe muscle pain involving all extremities.

He related recent diagnosis of dermatomyositis, taking several drugs and still rapidly worsening (as early as 1/2012 his ANA was 1:1280 and aldolase 20.5 U/L (Ref < 8.1 U/L). A skin biopsy 12/2011 showed infiltration of lymphocytes and macrophages, with basement membrane thickening). He had been diagnosed with dermatomyositis on 8/2/12 after several misdiagnoses were made of “sun rash” and eczema. He had no family history of autoimmune disorders. The prognosis given by his conventional physicians was “poor”.

**Fig. 1:**
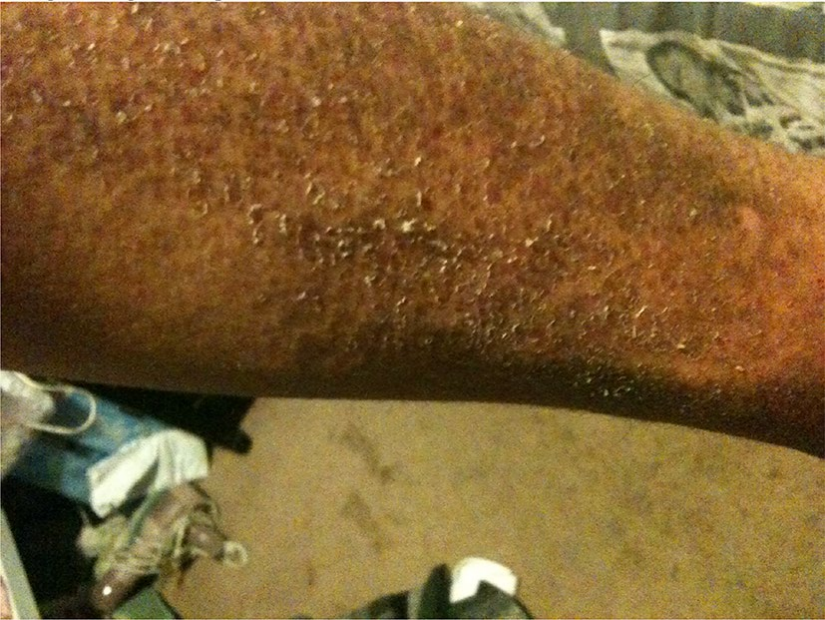
Leg of the patient before treatment

**Fig. 2:**
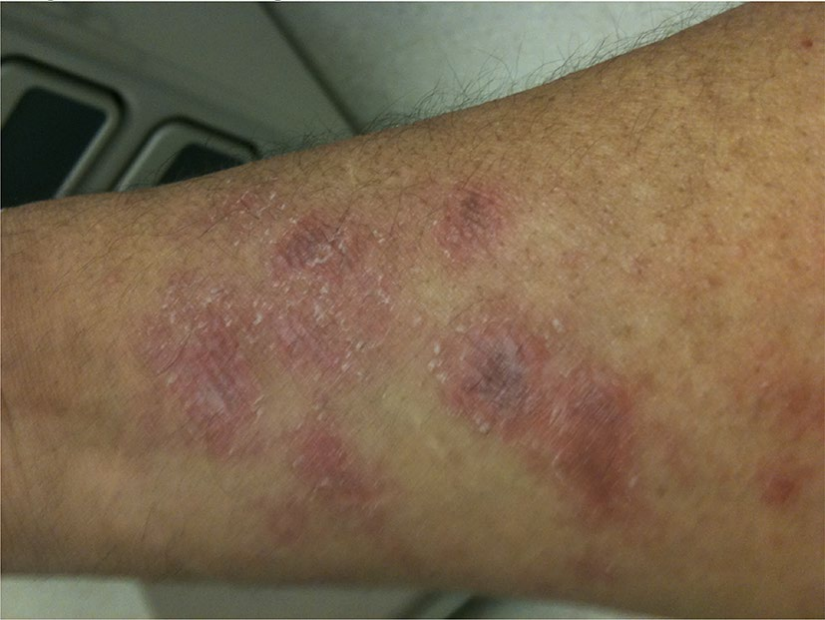
Forearm of the patient before treatment

**Fig. 3:**
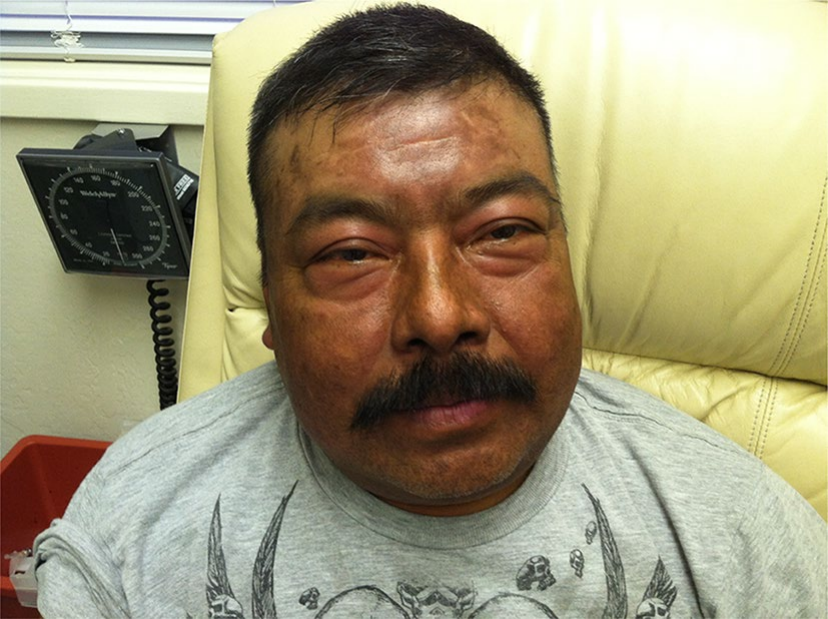
Face of the patient before treatment

He had quickly become extremely weak and found it all but impossible to do his field chores and earn a living. His skin had confluent brown macular rash with violet hue, almost ecchymotic on the extensor surfaces of the forearms and left ankle. He had an inflamed facial butterfly facial rash, moon facies, Gottron’s papules on the dorsal surface of hands, and nail cuticle telangiectasias. His muscles were very weak and tender. He exhibited classic signs of iatrogenic Cushing’s disease (Figs. 1, 2, 3).

Drugs for immunosuppression: prednisone 80 mg daily, methotrexate 10 mg/week, Plaquenil 200 mg twice daily, azathioprine 150 mg daily (methotrexate was discontinued after a short course due to spike in liver enzymes. AST rose to 130 on 28/8/12); acetaminophen/codeine twice daily for pain.

Over several months, his doctors repeatedly attempted weaning to 60 mg prednisone. However, every time he reached that level, his disease sharply flared. In March 2012, his CK was 2088 (44–96 U/L), CK (MB) 103.99, ANA 1:128, ESR 1. On 6-6-13, CK peaked at 9293 U/L as his treating physicians struggled to control his deteriorating condition with immunosuppressive drugs. Prednisone dose was intermittently raised to 120 mg/daily. I was not involved in any aspect of his drug regimen (until weaning after his dental work was completed).

Additionally, early in his disease, he was treated for *Helicobacter pylori* receiving amoxicillin, metronidazole, bismuth subsalicylate, and proton pump inhibitors. He also had a course of INH and rifampin for “latent” tuberculosis during this period, without change in the dermatomyositis.

Based on history and physical examination actually performed in his agricultural workplace, I suspected underlying and asymptomatic dental infections and sent him for dental X-rays of incisors and an extraction site. I also immediately started ozone therapy (described below) after obtaining full informed consent.

Dental X-rays confirmed my suspicions: failing endodontics at numbers 7, 8, and 9, and osteonecrosis at extraction site 32. I suggested that he have the infected teeth removed, but he refused through May 2013. However, by then he was up to 120 mg prednisone and his field labor was made possible by use of ozone therapy.

In July 2013, he finally underwent extraction of the failing root canals at 7, 8, and 9, with limited improvement. I suspected more subclinical dental infections and implored him to seek further treatment. However, cost was a major factor. He finally had more definitive dental work (fall 2014) and had teeth 4, 15, and 30 extracted. Following this, in conjunction with ozone therapy, I weaned his prednisone without incident or exacerbation (about 5 mg every other week). Prednisone was discontinued by 1/15. Plaquenil was discontinued the following month (2/15). He suffered no skin or muscle symptoms during this period or any clinical signs of inflammation.

His ozone therapy was as follows:

HBO_3_ beginning June 12, 2012 for a total of 13 treatments through 3-13-15 as follows:

- 6-12-12: 12,000 mcg; 2-2-13: 24,000 mcg; 10-15-13: 9000 mcg;
- 10-17-13: 9000 mcg; 1-11-14: 14,000 mcg; 5-10-14: 28,000 mcg;
- 8-2-14: 28,000 mcg; 11-13-14: 14,000 mcg; 3-13-15: 28,000 mcg;
- 8-1-15: 28,000 mcg; 3-18-16: 28,000 mcg; 6-24-16: 28,000 mcg;
- 1-17-17: 28,000 mcg (dates: month-day-year).

Ozone treatments improved his strength, vitality, and pain virtually immediately, even before the removal of oral infections, but results were partial only. Ozone therapy did appear to be the key factor in keeping him productive in the field, earning his living, during the entire period. He had been rapidly deteriorating clinically on drug therapy alone and was unable to work until receiving ozone therapy.

He also received DIV ozone averaging once every 2 weeks for a total of 30 sessions between 11/19/14 and 4-27-15. This consisted of 20 cc of O_2_/O_3_ gas at 25 mcg O_3_/cc.

Plaquenil was discontinued in 2-15. He continues to receive HBO_3_ therapy quarterly as a courtesy in ozone training classes. He remained totally asymptomatic until March 2017, telling me every time I saw him (weekly) “I never felt better”.

**Fig. 4:**
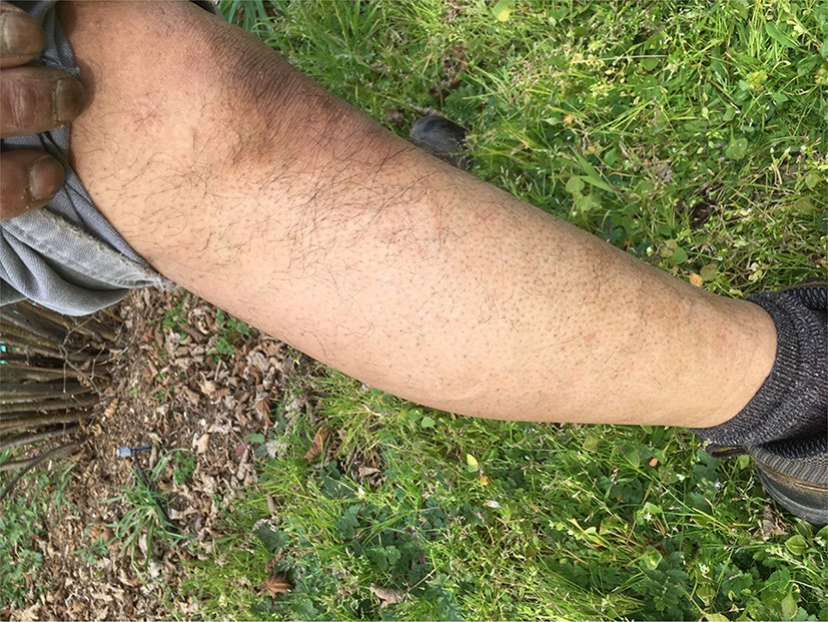
Leg of the patient after treatment

**Fig. 5:**
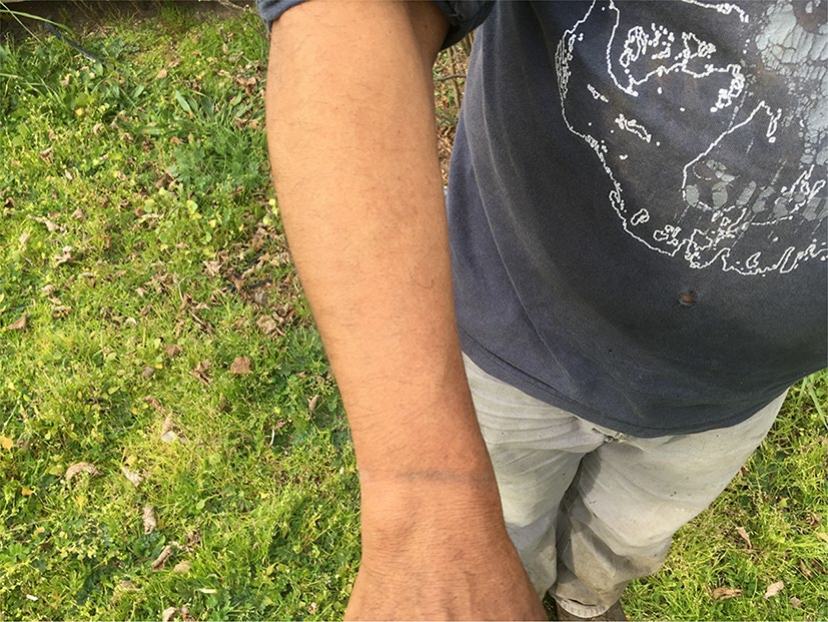
Forearm of the patient after treatment

**Fig. 6:**
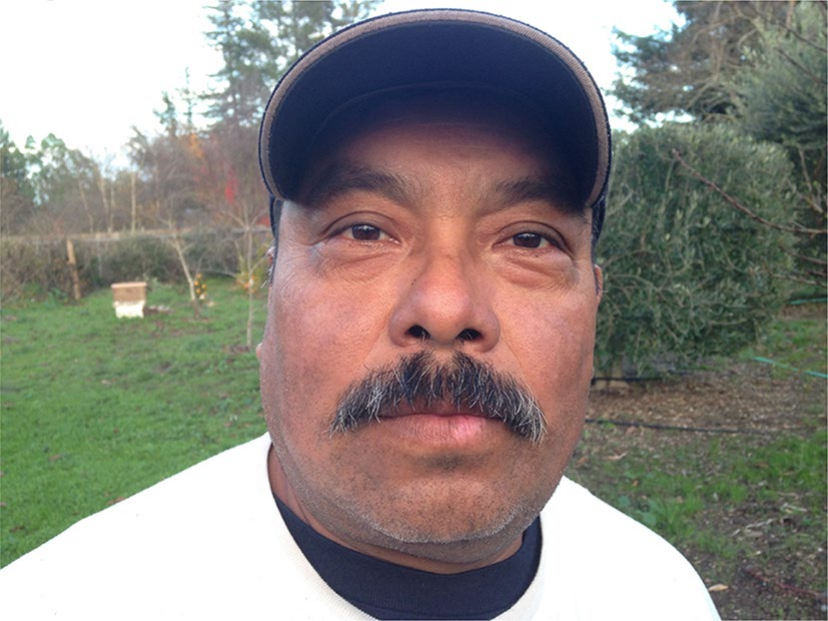
Face of the patient after treatment

In March 2017, he noted slight achiness with faint skin blotches. His CK (3/2/17) was 523 U/L. His doctors were prepared to restart the powerful immunosuppressive drugs. My examination suggested at least two more occult infected dental sites. I suggested he return to his dentist. Within 2 weeks, his dentist removed two more infected teeth, numbers 2 and 15 and his symptoms were gone virtually immediately. On June 10, 2017, his CK had dropped to 313 and he was again “never feeling better”. He suffered no adverse of unanticipated events (Figs. 4, 5, 6).

## Discussion

Oral infection, including occult cavitations and endodontically treated teeth, may be key sources of bacterial seeding in the body that is routinely overlooked. Weston Price, DDS, a renowned researcher of the last century, published his findings on the systemic risks of endodontic infections [9]. He found that he could transfer human diseases (from arthritis to carditis) to a rabbit by implanting a fragment of the sick person’s extracted endodontically treated tooth into the belly area of rabbits. Anaerobic organisms were difficult to culture in his day. Transplanting the infected structure and observing ramifications were his means of demonstrating transmissible agent. Dr. Price and his research team found that the implanted tooth from a human to an animal would induce the same systemic complications in the animal as were suffered by the human.

This case also supports long held concerns about the systemic effects of oral infections. Chan, et al. described a mechanism of post-infection autoimmunity: “postinfection production of cross-reactive tissue-specific autoantibodies” [10]. There have been reports of infection-mediated “autoimmune” diseases for decades [11–13]. Endodontic infections have been reported to harbor a plethora of toxic organisms [14, 15]. Modern research supports the older research of Price linking oral pathogens to systemic diseases. Mougeot et al. reported that *Porphyromonas gingivalis* was the most abundant species detected in coronary and femoral arteries [16]. Oral species have recently been found in the prostatic fluid of men with chronic prostate disease [17]. It is therefore more than reasonable to consider that inflammation in any organ/tissue (inclusive of “autoimmune” conditions) could be secondary to occult infection and certainly with oral organisms as the potential culprit. It is also reasonable to assume the “distant” manifestations (inflammation) will not clear until the seeding has ceased and/or the primary infection is eliminated which could relieve the body of production of microbe targeted but cross-reactive antibodies and/or dissemination of toxic microbial products. The patient’s flare in March 2017, and resolution with subsequent dental work, demonstrates that there can remain offending organisms after a primary pathology removal, which, if left unattended, could trigger relapse.

Additionally, German brothers, Huneke, described relationships between specific teeth and acupuncture meridians/organs, and joints to which they are related [18]. They proposed that disturbances in teeth (as well as scars) could cause distant body pathology via autonomic nervous system disturbances. Addressing these could immediately free the autonomic nervous system’s abnormal reflex. With this patient, I performed a “Huneke” test by injecting German described odonton points (in the gum on the buccal side of the suspect tooth) with just 0.3 mL 1% procaine, in the field. He was immediately relieved of his debilitating systemic pain which had persisted despite massive immunosuppressive drugs. He was able to work the rest of the day (no change observed in the rashes). Such a rapid phenomenon, not necessarily curative but pathology indicative, was coined “the lightning reaction” by Hunekes. This patient’s improvement lasted 48 h and caused him to act on my urgings to see a dentist. Immediately after his dental infections were finally removed, I was able to, relatively rapidly, steadily taper and discontinue his high-dose prednisone, and stop Plaquenil 1 month later.

Ozone therapy, in continuous worldwide use for 150 years, is a modality that antedates the modern “FDA approved” category of therapies. “Its effects are proven, consistent and with minimal side effects…. and well documented”, for infections, wounds and multiple diseases [19]. Under US law, licensed practitioners may modify and use ozone devices solely in their practice [20].

Regarding ozone therapy, several reports have emerged demonstrating ozone therapy’s ability to significantly reduce inflammatory mediators and joint damage [21, 22]. Fahmy’s book discusses in-house clinical results with ozone on rheumatoid arthritis showing significantly reduced pain, swelling and increased function [23]. A Brazilian paper presented at the American Academy of Rheumatology Meeting reported very significant improvement in joint pain and function in patients receiving ozone gas into their osteoarthritic joints [24].

Ozone therapy has other highly desirable circulatory and immunological effects. Basic published research (scores of articles) from Italy [25] and Cuba [26] has led to publication of books containing their peer-reviewed published studies. Both research teams have independently confirmed: (1) immune system modulation balancing its inflammatory/anti-inflammatory cytokines, (2) increase in production of RBC 2, 3 DGP (greater oxygen release) and improved rheology properties of blood (increased RBC flexibility), (3) elevation of key anti-oxidant enzymes such as SOD and increased glutathione achieving a redox cell balance. Menendez group has confirmed modulation of TNF-a by ozone therapy preconditioning in animals to be on a par with injected steroids [27].

All of these effects would be expected to positively influence the course of any disorder. The immune modulating effects would be particularly beneficial in inflammatory conditions. Ozone therapy has not been reported to induce harm, dependence, injury or untoward effects when properly administered.

Ozone therapy has significant activity against microorganisms. It nearly instantly destroys pathogens exposed to the gas. Scripps recently discovered ozone synthesis in the body [28] as part of its complex oxidative defense armamentarium. My group used DIV ozone in successfully and rapidly remitting five of five cases of acute Ebola in Sierra Leone during the 2014 epidemic, which otherwise had a 60% mortality rate [29]. Cuban researchers show that simply preconditioning animals with ozone therapy significantly reduces mortality to an intraperitoneal implant of fecal material [30] and significantly improves effectiveness of the antibiotic combination tazobactam/piperacillin in sepsis survival [31]. They also report modulation of TNF-alpha production in animals on par with dexamethasone [27].

Ozone gas and ozonized water cut through biofilms [32], well established as source of pathology (plaque) in the oral cavity, and is widely used in dentistry.

DIV oxygen has long been administered in Europe, found to stimulate the production of the anti-inflammatory enzyme 15-Lox-1 from eosinophils [33] and improve prostacyclin/thromboxane ratios [7].

The HBO_3_ method of ozone delivery may have a similar intravenous O_2_ gas like effect, in addition to the ozone effect itself. Boyle’s law of gas diffusion into fluids predicts about 8.8 cc of oxygen gas dissolved into the 200 cc blood by the HBO_3_ method described, which may well cause a “Regelsberger” effect [6].

This patient credits ozone therapy keeping him able to earn a living while in the long process of resolving the dental issues. Neither ozone therapy nor any drug therapy would be expected to singularly remedy closed foci of infection, such as in this case, essentially abscesses.

CK values need discussion. In a review article on CK levels, the authors argue that the current accepted “normal” levels of CK are low. CK levels vary by sex, race, body mass and physical activity [34]. “While the current reference range is commonly held at 200 IU/L, the European Federation of Neurological Societies suggests redefining elevated CK as values 1.5 times beyond the upper limit of normal”. The laboratory that performed this patient’s initial CK limit was 98 IU/L. The review article maintains that based on two standard deviations threshold, a level up to 504 in white men (higher in Africans) is acceptable. Clearly, the patient’s CK levels in the thousands would be universally held as pathological. His June 2017 level of 313 IU/L appears quite normal for a muscular field laborer of Mexican extraction according to this reference.

“Spontaneous remission” is highly unlikely as every effort to lower immunosuppressive drugs was met with failure (severe exacerbation) until immediately after dental attention and receiving ozone therapy, permitting steady and uneventful withdrawal of long-term high-dose steroids, which would have fully dampened his pituitary–adrenal axis.

The strength of this case is that it showed high value in addressing a medically unsuspected cause of systemic disease (occult dental infection) and in tailoring a specific treatment plan to the individual patient and not the disease model. The limitation is that autoimmune disease likely has multiple causes (such as allergy, toxin induced, genetic) and the next case may be totally different. It is important to go beyond a conventional history and physical examination, which may arrive merely at a descriptive diagnosis, to elucidate the cause of the malady.

This author has seen many “unrelated” systemic or distant symptoms remit when suspect endodontically treated teeth were removed or jaw cavitations surgically treated. With the modern discoveries of oral bacterial products present in distant inflamed tissue, and the discoveries of alimentary canal organisms in inflamed tissue, medicine/dentistry should revisit the research of the pioneering Weston Price, DDS, to examine undiscovered links between teeth, the oral cavity and the body.

## Patient perspective

This patient has publicly expressed extreme gratitude for his recovery by the combination of the two approaches—dental treatment and ozone therapy—after he rapidly deteriorated while on aggressive chemical therapy. At the outset, he was incapable of performing his field duties. After treatment, he consistently related, “I’ve never felt better.”

## Conclusions

This report appears to be the world’s first describing total resolution of an autoimmune disease in crisis with removal of oral infection complemented by ozone therapy. It re-presents to the medical world a critical, overlooked and relatively unknown factor and treatment approach in the management of chronic inflammation—occult dental infection—to which both the medical and dental professions must become aware.

Inflammatory conditions are notoriously difficult to treat, often requiring highly toxic drugs. This report is also valuable across the board in medicine, bringing forward a novel and non-toxic therapy (ozone) as an alternative and/or adjunctive treatment to drugs. Ozone therapy could advance medicine’s currently limited ability to safely treat inflammatory (and infective) disorders. It is the author’s hope that this report will stimulate institutional study, which, in today’s modern medical world, may be difficult due to ozone’s non-patentability for financial returns.

## Abbreviations

Dates: Day/month/year
CK: Creatine kinase
DIV: Direct intravenous gas
MAH: Major autohemotherapy
HBO_3_: Hyperbaric ozone therapy
